# Sous-vide processing of silver carp: Effect of processing temperature and cold storage duration on the microbial quality of the product as well as modeling by artificial neural networks

**DOI:** 10.1371/journal.pone.0246708

**Published:** 2023-03-29

**Authors:** Seyed Vali Hosseini, Milad Pero, Zahra Hoseinabadi, Reza Tahergorabi, Shirin Kazemzadeh, Ricardo Santos Alemán, Jhunior Abrahan Marcia Fuentes, Ismael Montero Fernández, David P. Calderon, Xesus Feas Sanchez

**Affiliations:** 1 Department of Fisheries, Faculty of Natural Resources, University of Tehran, Karaj, Iran; 2 Department of Food Science, Engineering and Technology, College of Agriculture and Natural Resources, University of Tehran, Karaj, Iran; 3 Department of Health and Food Safety, Faculty of Health, Qazvin University of Medical Sciences, Qazvin, Iran; 4 Food and Nutritional Sciences Program, North Carolina Agricultural and Technical State University, Greensboro, NC, United States of America; 5 Department of Food Science, Louisiana State University, Baton Rouge, LA, United States of America; 6 Technology Sciences Faculty, National University of Agriculture, Catacamas, Olancho, Honduras; 7 Department of Organic and Inorganic Chemistry, University of Extremadura, Cáceres, Spain; 8 Academy of Veterinary Sciences of Galicia, Edificio EGAP, Santiago de Compostela (A Coruña), Spain; Spanish Council for Scientific Research (CSIC), SPAIN

## Abstract

Silver carp (*Hypophthalmichthys molitrixi*) was processed by sous-vide method at different temperatures (60, 65, 70, and 75°C). Then, the microbiological quality of the processed samples was monitored during cold storage (4°C) for 21 days. The target microorganisms were Enterobacteriaceae, Lactic Acid bacteria (LAB), Pseudomonas, Psychrotrophs, and total viable count (TVC). In samples processed at 75°C, the presence of Enterobacteriaceae, Pseudomonas and Psychrotrophs were not detectable up to 15 days of storage and lactic acid bacteria were not detectable even at the end of the storage period. A radial basis function neural network (RBFNN) model was established to predict the changes in the microbial content of silver carp. In this step, the relationship between processing temperature and storage duration on microbial growth was modeled by ANNs (artificial neural networks). The optimal ANN topology for modeling Enterobacteriaceae, Pseudomonas, and Psychrotroph contained 9 neurons in the hidden layer, but it contained 15 and 14 neurons for TVC and LAB, respectively. By experimenting with the temperature of -80°C, it was revealed that the obtained ANN model has a high potential for prediction.

## Introduction

Silver carp (*Hypophthalmichthys molitrix*) is a popular fish in the world due to its desirable properties such as high nutritional content, fast growth, high feed efficiency, and easy cultivation. As a result, it is the most cultured fish in the world. Silver carp have been used to control the growth and removal of algal blooms [1]. Unfortunately, the food safety and spoilage of fishery products are a huge concern because of microbial cross-contamination from various sources. Moreover, because of its neutral pH and high water content, the shelf-life of fish declines rapidly. Thus, there is a need to increase the shelf life of fishery products.

Sous-vide is a French word meaning “under vacuum”, and sous-vide cooking is a processing method in which vacuum-packed foods in heat-resistant pouches are cooked under controlled temperature and time [2]. The advantages of sous-vide cooked products are that these products are not preserved by low water activity or pH and do not contain preservatives. The safety of these types of products relies on high heat treatment and cold storage. On the other hand, the increasing demand of consumers for safe but minimally processed fresh fisheries products, together with the increasing concerns about the use of certain chemical preservatives, has led to a growth in the use of sous-vide processing technology to extend the shelf life and maintain the quality of fresh fisheries products [3]. In addition, there is no chance for recontamination as the sous-vide process takes place after vacuum packaging. Furthermore, the temperature of heating is not that high to affect the original flavor, texture, and nutritional qualities [4].

In this technique, the products are immersed in a hot water/steam oven for a longer time than conventional cooking followed by immediate cooling) lower than 4°C) [5]. Recently, sous-vide processing was applied and combined with irradiation for mackerel fillets [6]. They concluded that the microbial count of mesophilic and psychrophilic bacteria treated by sous-vide and irradiation never exceeded the standard limit.

Modeling and optimization play an important role in the reduction of waste, product quality improvement, and profitability. The application of mathematical models has the advantages of predicting results and process optimization without the need for conducting experiments [7]. Owing to the ability of predictive modeling in the improvement of food safety, it has gained a great interest among food industries [8]. There are many modeling and optimization techniques applied in food technology [9], but among them, artificial neural networks (ANNs) have gained special attention This modeling technique can be used for optimization in the food industry, due to their ability in modeling complex processes where there is a nonlinear relationship between the dependent and independent variables [10]. ANNs can model dynamic, nonlinear phenomena that are too complex to be described by simple combinations of analytical properties or by empirical rules or when relationships between cause and effects are uncertain. Therefore, they can be applied for either diagnosis or predictions [11]. ANNs mimic the human brain in order to explain complex phenomena with the ability to manage imprecise information and generalize the processes [12]. Furthermore, this modeling technique has successfully been applied in the modeling and optimization of food processes [13–15] and microbial analysis [16–19].

To the best of our knowledge, there is no study about the effects of sous-vide processing on the microbial quality of silver carp during cold storage. Therefore, the main objective of this study was to analyze the effect of sous-vide processing conditions on the microbial quality of silver carp. Moreover, the effects of these processing conditions on microbial quality were modeled by ANNs as a powerful modeling tool for complex relationships. The steps of this research were as follows: (1) sous-vide processing of silver carp at different process conditions; (2) analyzing the effect of process conditions on the microbial quality (Enterobacteriaceae, Pseudomonas, Psychrotrophs, lactic acid bacteria, and total viable count) of silver carp; (3) behavior of microbial growth in sous-vide cooked fish during cold storage (21 days); (4) modeling and simulation of microbial count by artificial neural networks.

## Material and methods

Silver carp samples (average weight and length of 790±20 g and 283±16 mm, respectively) were obtained from a local warm-water fish farm located in Rasht, in the north of Iran. The fish were not starved and were fed with commercial fish feed from the Faradaneh Company, Tehran, Iran. All microbial media such as Plate count agar (PCA), Pseudomonas Agar Base, Violet red bile glucose agar, Man Rogosa Sharp Agar medium cultures were supplied by HiMedia (HiMedia Co., India).

### Sample preparation

Sample preparation was done according to the procedures described by Orawan et al. (2016) [20]. Briefly, fish samples were cut into fillets with dimensions of 10×5×2 cm. Then, each fillet was vacuum packed by a vacuum packaging machine (Guater Control Back Co, Iran) with 95% of vacuum.

The bags used for this purpose were polyamide bags (S-Gruppen, Vinterbro, Norway) with 75 μm thickness and an oxygen transmission rate of 30 cm^3^ m^-2^ day^-1^ atm^-1^. Vacuum-packed samples were cooked at 60, 65, 70, and 75°C for 15 min in a water bath (Memmrt Co., Schwabach, Germany). After cooking, they were immediately immersed in an ice bath and then stored in the refrigerator (4°C) for 21 days [21].

### Microbial analysis

Microbial analysis was carried out on both control and treated samples at 0, 3, 7, 14, and 21 days of cold storage (4°C). At each storage interval, 5 g of sample was aseptically removed with the aid of a sterile scalpel and placed in a stomacher bag. 45 mL of sterile peptone saline solution (4°C) was poured into the stomacher bag containing the sample and the mixture was homogenized using a stomacher.

Plate count agar (PCA) was used for determining the total viable count by incubating at 37°C for 48 h. Pseudomonas count was obtained by culturing in Pseudomonas Agar Base according to the procedure described by the American Public Health Association [22]. Violet red bile glucose agar was used for Enterobacteriaceae count as described by Moini et al. [23]. For the determination of lactic acid bacteria, de Man Rogosa Sharp Agar was applied [23]. Psychrotrophs were determined on PCA followed by incubation at 4°C for 10 days [22].

### Artificial neural networks (ANNs) modeling

**Fig 1:**
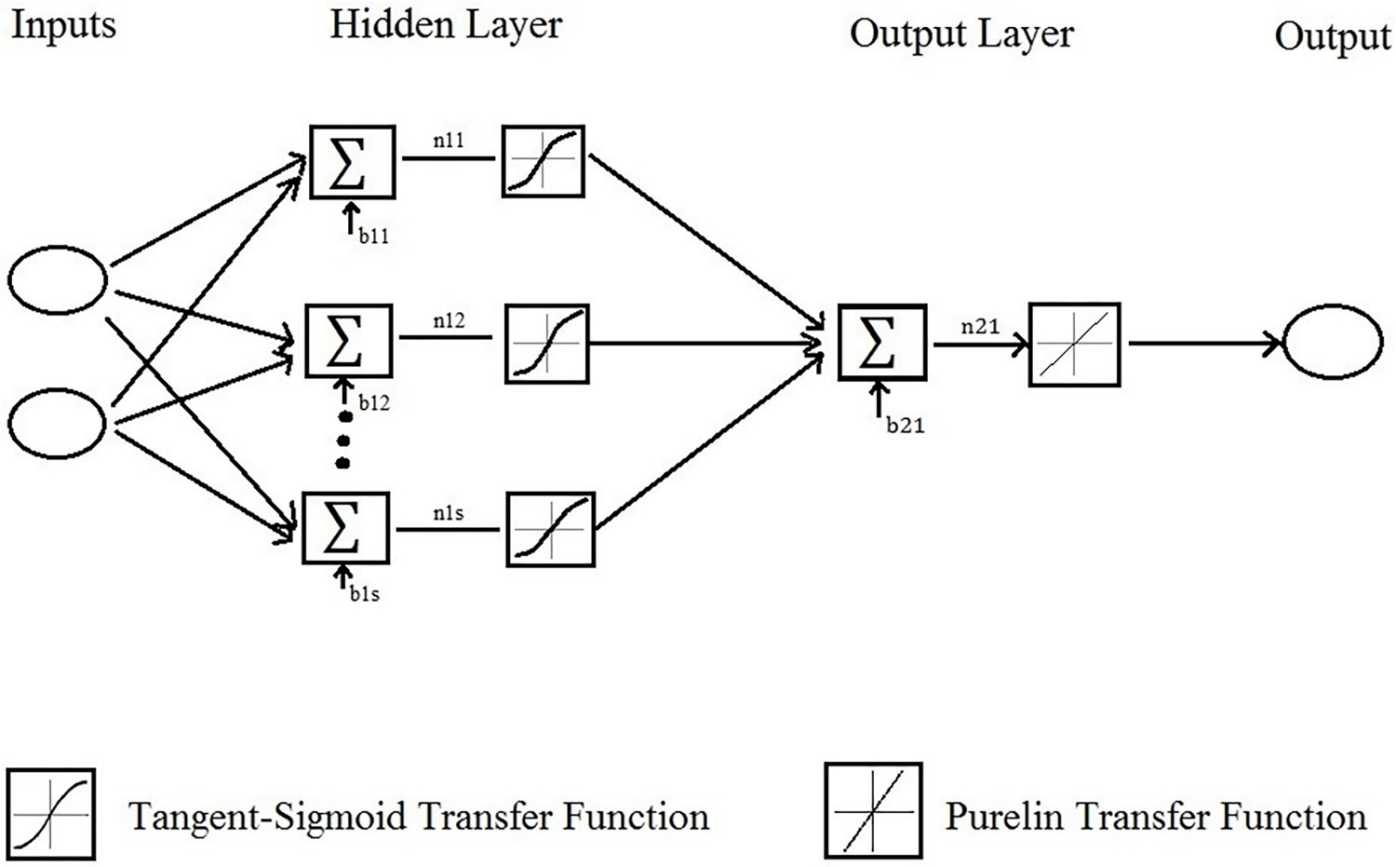
Structure of MLP ANN for modeling the microbial count. n, neuron; s, number of neurons; b, bias.

The experimental data used for developing ANNs were independent variables including cooking temperature (°C) and storage period (day) and the dependent variable was the microbial count (Log CFU g^-1^). For developing ANNs, 70, 15, and 15% of experimental data were randomly selected for training, cross-validation, and testing, respectively. Multilayer perceptron (MLP) and ANNs were used for modeling the microbial count (Fig 1). In the procedure of finding the best ANN structure, a different number of neurons (1 to 15) in the hidden layer were analyzed. For each microorganism, a separate optimal ANN was developed. The training algorithm of ANNs was Levenberg-Marquardt (LV) backpropagation (BP). The transfer function in the hidden layer was Tangent-Sigmoid (tansig) and for the output layer, was linear. Three replicates were carried out for each experimental run. The experimental run was a combination of cooking temperature and storage period [13]. Two statistical error criteria were taken into account for checking the goodness of fit for each ANN topology. These criteria were the coefficient of determination (R^2^) and root mean square error (RMSE):

$$R^{2}=1-\frac{\sum_{i=1}^{N}{\left(y_{pre}^{i}-y_{exp}^{i}\right)}^{2}}{\sum_{i=1}^{N}{\left(y_{pre}^{i}-\bar{y}\right)}^{2}}$$ (1)

$$RMSE=\sqrt{\frac{1}{N}\sum_{i=1}^{N}{\left(y_{pre}^{i}-y_{exp}^{i}\right)}^{2}}$$ (2)

where, y_pre_ and y_exp_ indicate the predicted and experimental dependent variable (microbial count), respectively. ӯ indicates the average of the experimental dependent variable and N is the total number of runs. Based on the maximum R^2^ and minimum RMSE, the best ANN topology was selected. R^2^ is scaled between 0 and 1, therefore, it can be easily interpreted, whereas RMSE is not scaled to any particular values. R^2^ is a relative measure of fit, but RMSE is an absolute measure of fit. Lower values of RMSE indicate a better fit. RMSE is a good measure of how accurately the model predicts the response, and it is the most important criterion for fit if the main purpose of the model is prediction. In general, higher R^2^ usually corresponds to lower RMSE but this is not always true and sometimes contradiction may occur. In this case, RMSE is considered as the determinative criterion. MATLAB (version 2013a, MathWorks, Massachusetts, United States) software was employed in ANN modeling (the Matlab code of present work has been provided as S1 File). The capability of the obtained ANN model in the prediction of the experiment at a temperature outside the range of modeling was investigated by experimenting with 80°C.

### Statistical analysis

On each sampling occasion, a minimum of three observations was collected unless specified otherwise. Descriptive statistics of analysis results were calculated for each treatment. All data were initially evaluated by analysis of variance (ANOVA). The data were tested for homogeneity of variances at a significant level of P <0.05 and a probability value less than 0.05 was considered statistically significant (Duncan, 1955). Microsoft Windows Excel 2010 and SPSS software (Version18.0, SPSS Inc., Chicago, USA) were used to analyze the data.

## Results and discussion

### Part 1: The experimental data analysis

**Fig 2:**
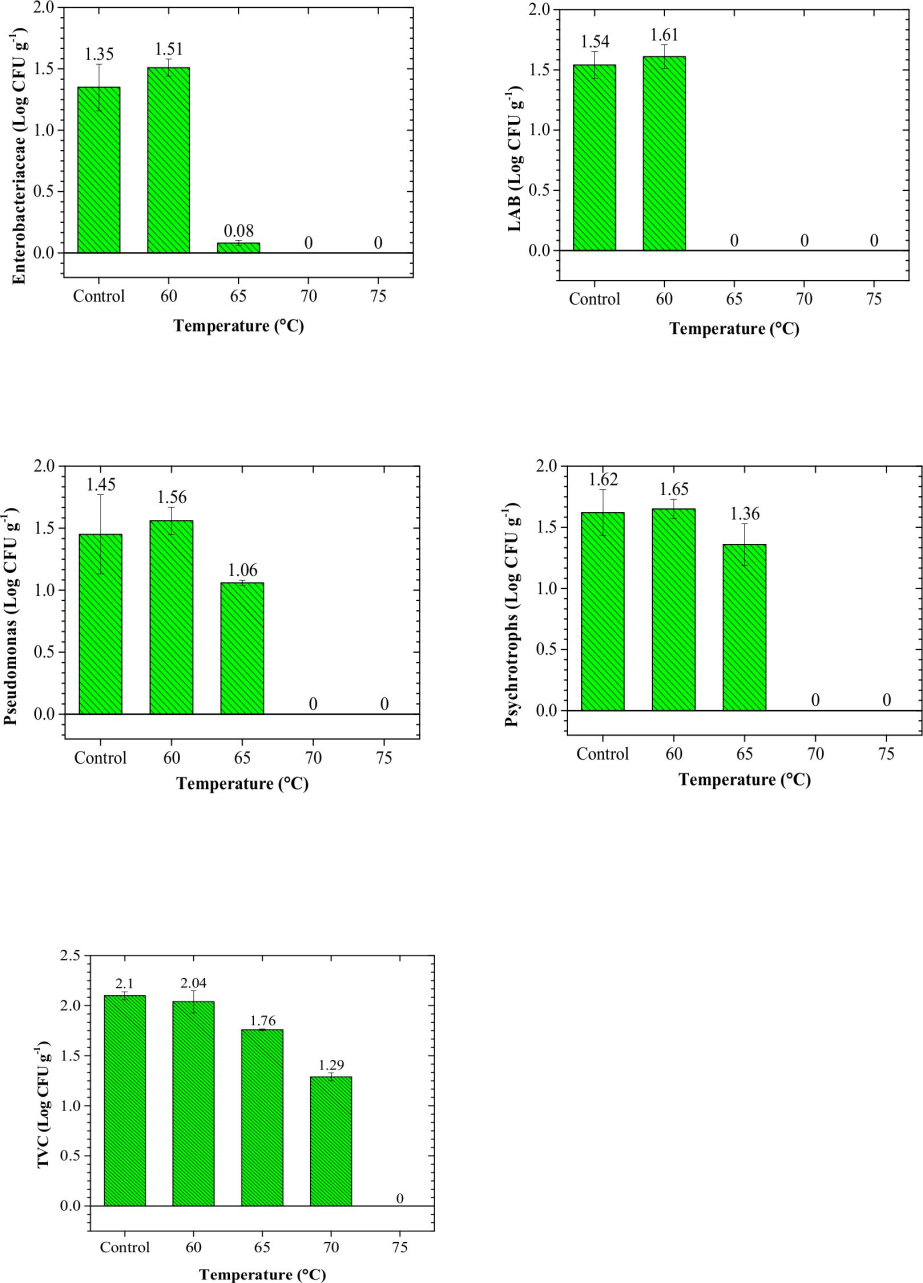
Results of microbial count (Enterobacteriaceae, Lactic Acid bacteria (LAB), Pseudomonas, Psychrotrophs, and total viable count (TVC)) of control and processed samples at time 0 (the day of processing).

**Fig 3:**
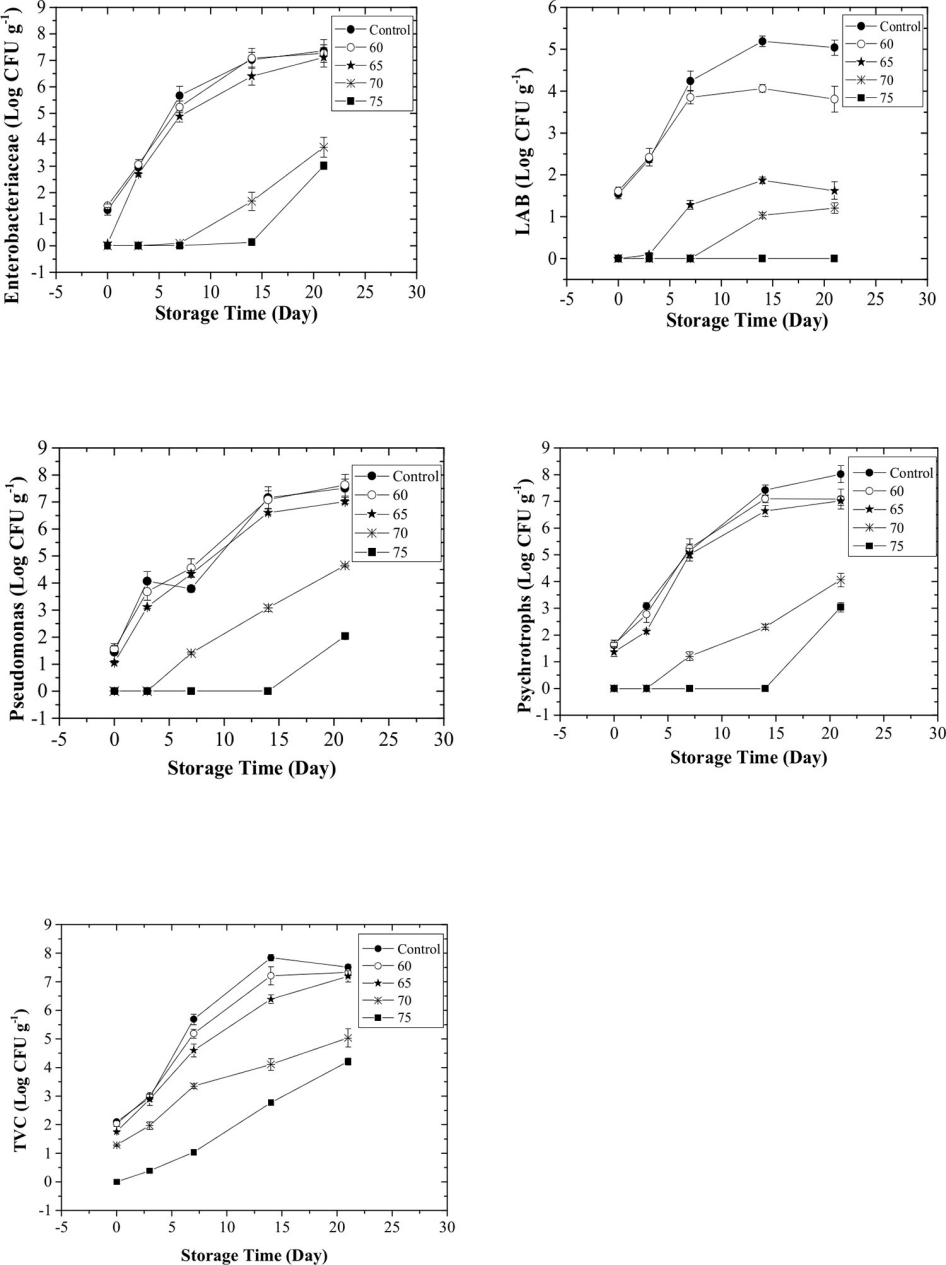
Growth kinetics of Enterobacteriaceae, Lactic Acid bacteria (LAB), Pseudomonas, Psychrotrophs, and total viable count (TVC) in control and processed samples during cold storage.

The effect of the processing temperature on the survival of microorganisms at time 0 (the day of processing) is seen in Fig 2. For total viable count (TVC), the critical temperature in which the bacterial count was undetectable was 75°C. There was not a significant difference (P>0.05) between control and samples processed at 60°C in terms of the bacterial load. For lactic acid bacteria (LAB), the process temperature of 65°C was considered as the critical temperature while this value for Enterobacteriaceae, Psychrotrophs, and Pseudomonas was 70°C. Similarly, the process temperature of 60°C was not effective in the inactivation of LAB, Enterobacteriaceae, Psychrotrophs, and Pseudomonas. Fig 3 shows the rate of microbial growth in treated samples during storage at 4°C. Although the critical temperature for TVC was 75°C, microorganisms started to grow in samples treated at this temperature and the final population reached about 4 log CFU g^-1^ after three weeks of storage. But, according to the recommended limit of TVC which is 7 Log CFU g^-1^, samples treated at this temperature are still in the range of acceptable limits. This is also true for samples treated at 70°C. The microbial load could be influenced by the *P*. *psychrophila*, *A*. *allosaccharophila*, and *S*. *putrefaciens*, which may be the most abundant bacteria as reported [24] from spoiled Silver carp samples. The rate of Enterobacteriaceae growth in samples treated at 65°C was very high, probably due to the incomplete inactivation of these microorganisms at this temperature. Even at higher process temperatures, the population of Enterobacteriaceae reached about 4 log CFU g^-1^ after three weeks of storage at cold temperatures. However, the presence of Enterobacteriaceae was not detectable in samples treated at 70°C after one week (critical temperature for Enterobacteriaceae) and this period was two weeks for samples treated at 75°C. These results are not far from those reported in common carp (*Cyprinus carpio*) where Enterobacteriaceae were detected after 14 days of storage in treated samples with sauce (30% tomato paste, 20% lemon juice, 30% oil, 10% garlic, 4% water, 3% salt, 1% red pepper, 1% cumin, and 1% thyme) and steam oven (90°C, 15 min). LAB did not grow in samples treated at 75°C even after three weeks of storage [25]. But for samples processed at the critical temperature of these microorganisms, the final population reached about one log CFU g^-1^ at the end of the storage period. These results are contradictory to some studies that detected LAB bacteria in considerable amounts at very high temperatures (90°C) using sous-vide processing technology in Common carp (*Cyprinus carpio*) [24]. Similarly, a considerable growth of LAB bacteria in vacuum-packaged Silver carp (*Hypophthalmichthys molitrix*) fillets was seen at 80 and 98°C. Psychotropic and Pseudomonas were not detectable in samples treated at 75°C after two weeks of storage, however, their final population reached 3 and 2 log CFU g^-1^ at the end of the storage period, respectively. The growth of TVC, psychrotrophic, pseudomonas, and Enterobacteriaceae in samples treated at high temperatures (75°C) could be associated with the thermolabile characteristics of these bacteria [26]. As reported, the growth of TVC in samples treated at high temperature (80 & 98°C) was predominant in salted and vacuum-packaged Silver carp (*Hypophthalmichthys molitrix*) fillets during storage [27]. Besides, TVC showed more heat resistance than Enterobacteriaceae in sous-vide cooked salmon loins processed at high pressure [5]. The overall results of microbial quality indicate that sous-vide processing of silver carp at 75°C was adequate for assuring the safety of samples for two weeks. As it is seen in Fig 3, the presence of microorganisms was not detectable at severe processing temperatures for a specific period (varied depending on the process temperature and type of microorganism) but, after that, they started to grow. This behavior was due to the recovery of thermally injured cells. The growing behavior of injured cells was different than viable cells in three ways: Firstly, the lag period of injured cells was increased considerably even using the optimum recovery medium. Secondly, the generation time of the injured cell can be longer than the total viable cells, even in the same intrinsic and extrinsic situations [28]. Thirdly, the recovery of injured cells was affected by the incubation temperature.

### Part 2: The artificial neural network analysis

**Fig 4:**
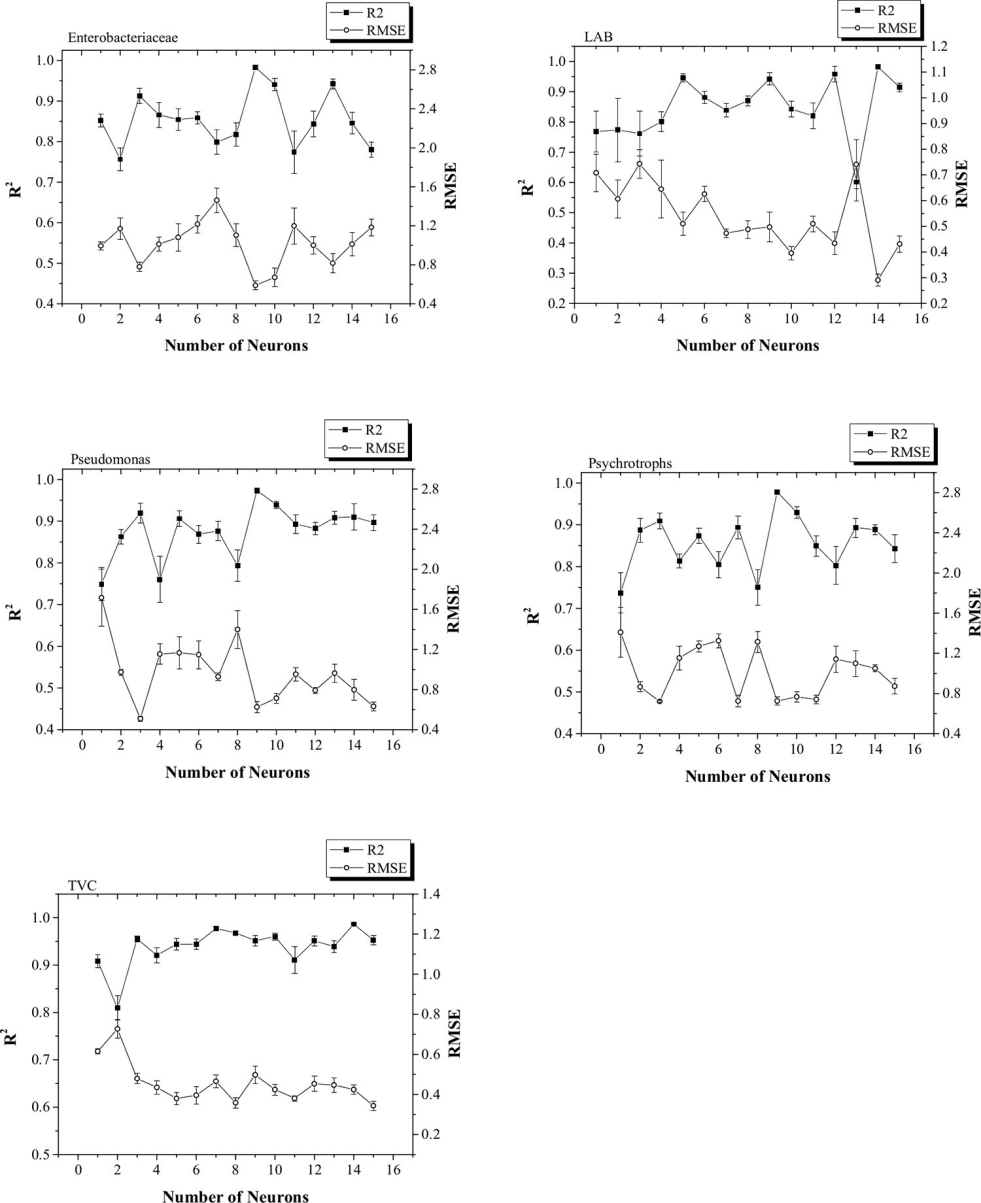
Average and standard error results of R2 and RMSE for five software runs in the procedure of finding the best number of neurons in the hidden layer of feedforward backpropagation ANNs.

In the next step, an artificial neural network (ANN) was employed to build the model of the microbial quality which was affected by the process temperature and storage time. Process temperature (°C) and storage time (day) were considered as independent variables and the microbial load of the bacteria was chosen as the dependent variable. For each group of bacteria, a separate ANN was developed. For developing an optimum ANN for the bacteria, a different number of neurons (starting from 1 to 15) in the hidden layer were determined. Therefore, 15 networks, each containing a variable number of neurons in the hidden layer (1 to 15) were analyzed. The accuracy of each network was monitored based on the R^2^ and RMSE. One of the important issues that should be considered in ANN modeling is that if it is obtained a specific result in the first run, it will not necessarily get the same result in the next replicate [14]. This issue is due to the random selection and allocation of the input data for each section of the network development process, namely training, cross-validation, and testing. Therefore, it is recommended to repeat the run and report the average R^2^ and RMSE for each network with a different number of neurons in the hidden layer. In this study, the runs were repeated five times, and, based on the average R^2^ and RMSE, the best ANN was obtained for each group of bacteria (Fig 4).

**Fig 5:**
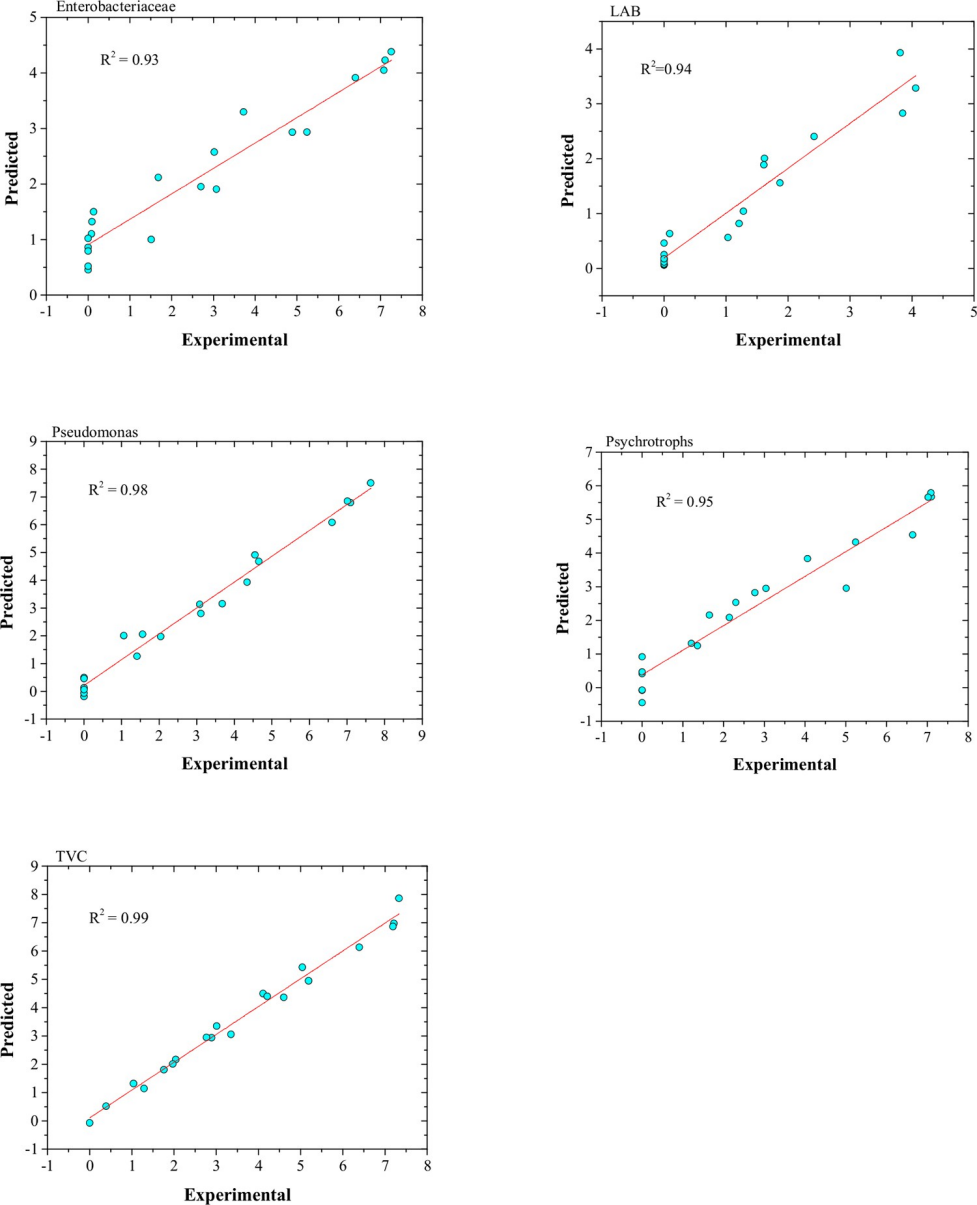
Comparison between predicted and experimental microbial count (Enterobacteriaceae, Lactic Acid bacteria (LAB), Pseudomonas, Psychrotrophs, and total viable count (TVC)). Predicted microbial counts were obtained by the best ANN topology.

For Enterobacteriaceae, Pseudomonas, and Psychrotroph the best ANN was obtained when the number of neurons in the hidden layer was 9, but, for LAB and TVC, this was 14 and 15 neurons, respectively. Fig 5 shows the correlation between predicted (by the optimum network) and experimental data. As it is seen, a high correlation was obtained for all sets of bacteria which indicates the capability of ANN in modeling and predicting the microbial quality of silver carp processed by the sous-vide technique. This is especially important in industrial applications where the determination of optimum process conditions and prediction ability results in reduced costs and therefore higher profit [17].

Pero et al. (2018) applied ANNs for modeling the effect of process temperature and duration and storage period on the color of broccoli [14]. Their study is similar to the present work in terms of modeling but the dependent variable was color. They reported that finding a relationship between process temperature and duration and storage period can be obtained easily by ANNs modeling. Therefore, they applied ANNs and the obtained result revealed the capability of ANNs in modeling such complex processes.

**Fig 6:**
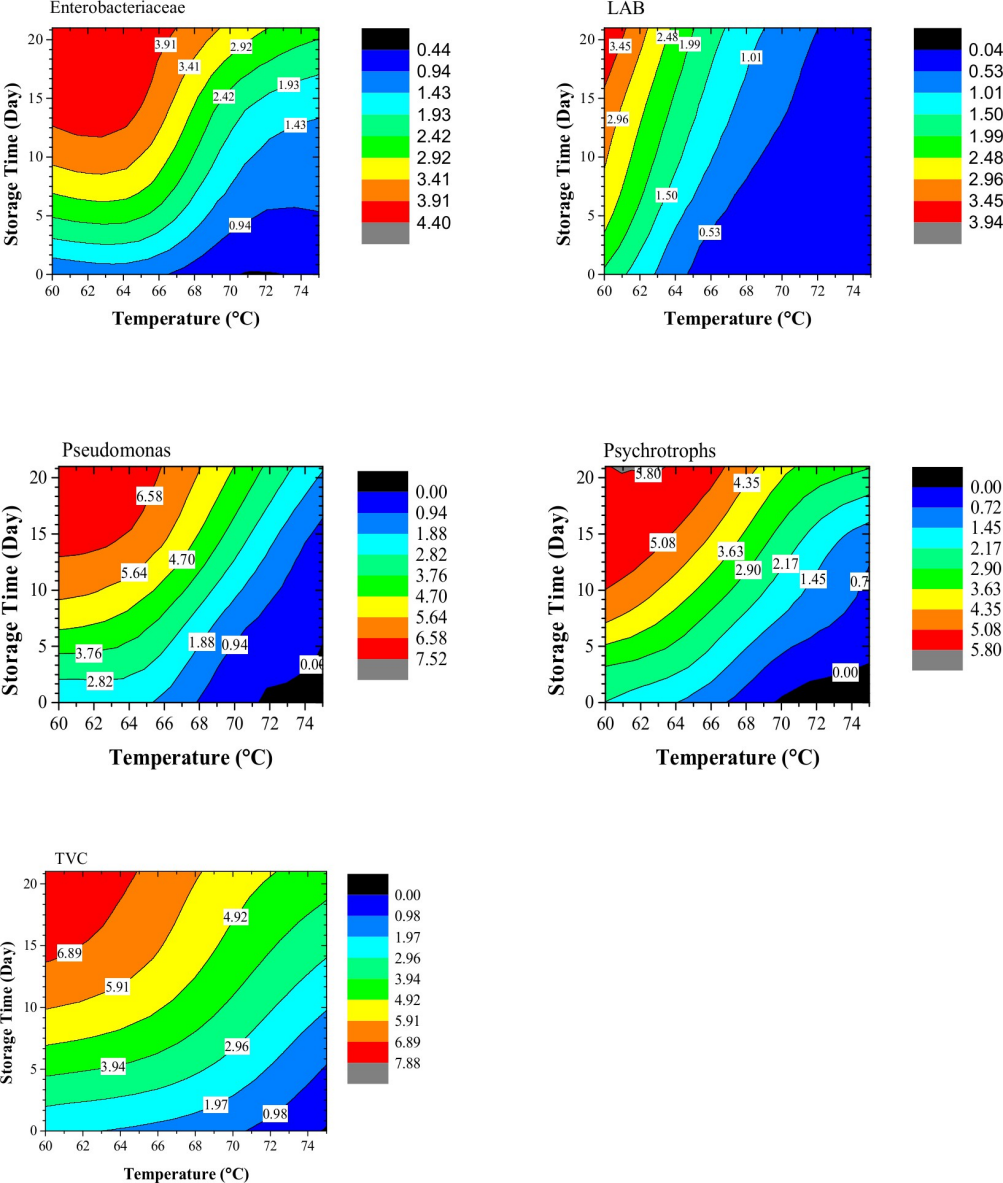
Simulated microbial count (Enterobacteriaceae, Lactic Acid bacteria (LAB), Pseudomonas, Psychrotrophs, and total viable count (TVC)) of sous-vide processed samples at different temperatures during cold storage.

Simulated results of ANN were used to generate the contour plots of microbial growth during storage (Fig 6). These graphs provide useful and comprehensive information, especially for optimization purposes. From these graphs, for each group of microorganisms, the microbial load in silver carp fillets processed at a temperature between 60 to 75°C and storage up to 21 days was obtained.

In order to check the prediction capability of the obtained ANN model, an experiment was conducted at 80°C, as it is seen in Table 1, the ANN model has a good power of prediction. This result indicates that ANN can be successfully employed for the prediction purposes of the sous-vide processing of the silver carp. This is important especially from an industrial point of view where accurate prediction of the process can reduce the cost and time.

**Table 1:** Experimental (Exp.) and predicted values of microbial count (Log CFU g^-1^) of sous-vide samples processed at 80°C.

Storage Time	Enterobacteriaceae (Log CFU g^-1^)		LAB (Log CFU g^-1^)		Pseudomonas (Log CFU g^-1^)		Psychrotrophs (Log CFU g^-1^)		TVC (Log CFU g^-1^)	
(Day)	Exp.	Predicted	Exp.	Predicted	Exp.	Predicted	Exp.	Predicted	Exp.	Predicted
0	ND	0.00	ND	0.00	ND	0.00	ND	0.00	ND	0.00
3	ND	0.00	ND	0.00	ND	0.00	ND	0.00	ND	0.55 ± 0.04
7	ND	0.02 ± 0.00	ND	0.00	ND	0.00	ND	0.00	0.94 ± 0.22	0.98 ± 0.17
14	ND	0.05 ± 0.01	ND	0.10 ± 0.02	0.53 + 0.20	1.02 ± 0.16	ND	0.28 ± 0.13	1.40 ± 0.09	1.89 ± 0.37
21	1.10 ± 0.22	1.40 + 0.13	ND	0.34 ± 0.41	1.32 ± 0.14	1.83 + 0.30	ND	0.62 ± 0.08	2.26 ± 0.08	2.92 ± 0.21

## Conclusion

This study reports the influence of sous-vide processing on the microbial quality of silver carp (*Hypophthalmichthys molitrixi*) by analyzing the microbial load of LAB, psychrotrophic, Pseudomonas, Enterobacteriaceae, and total viable count (TVC) affected by the processing temperature and storage period. The results illustrated that sous-vide processing at 75°C could significantly inhibit the microbial load of silver carp fillets. Moreover, the effects of process conditions and storage period on the microbial quality of samples were successfully modeled by artificial neural networks. This provides a powerful tool for manufacturers and researchers for the optimization of processes in which there is a complex relationship among variables. The simulation results of this research, presented by contour plots, provide a useful tool in optimizing the sous-vide processing of silver carp, and also they can be used in designing an experiment for their studies of silver carp processing. Based on the simulation results, processing at 75°C will cause the count of Enterobacteriaceae, LAB, Pseudomonas, Psychrotrophs, and TVC to be at 10% of their control sample after 5, 21, 15, 10 and 2 days of storage, respectively. The prediction capability of the obtained model was successfully validated by experimenting with -80°C. It should be noted that the obtained ANN model is only valid for storage temperature of 4°C and other storage temperatures, a new ANN model should be obtained.

## Supporting information

S1 File The Matlab code of present work has been provided as *“Supporting information”*. (RAR)
